# Accuracy of magnetic resonance imaging for diagnosing hallux valgus

**DOI:** 10.1590/0100-3984.2019.0054

**Published:** 2020

**Authors:** Paulo Victor Partezani Helito, Stephano Raydan Ramalho Rocha, Rafael Trevisan Ortiz, Giovanni Guido Cerri, Claudia da Costa Leite, Marcelo Bordalo Rodrigues

**Affiliations:** 1 Hospital Sírio-Libanês, São Paulo, SP, Brazil.; 2 Instituto de Ortopedia e Traumatologia da Faculdade de Medicina da Universidade de São Paulo (IOT-FMUSP), São Paulo, SP, Brazil.

**Keywords:** Magnetic resonance imaging, Foot, Hallux valgus, Forefoot, human/diagnostic imaging, Metatarsophalangeal joint/ diagnostic imaging, Ressonância magnética, Pé, Hálux valgo, Antepé humano/diagnóstico por imagem, Ângulo metatarsofalangiano/diagnóstico por imagem

## Abstract

**Objective:**

To assess the accuracy of magnetic resonance imaging (MRI) for the diagnosis of hallux valgus using radiography during weight bearing as the gold standard.

**Materials and Methods:**

This was a retrospective analysis of all patients undergoing MRI of the foot and radiography of the foot during weight bearing at our institution between January and June of 2015. The hallux valgus angle (HVA) was measured on MRI and radiography. The Wilcoxon signed-rank test and simple linear regression were used in order to compare measurements. Patients were divided into two groups according to the HVA determined on radiography: > 15° (hallux valgus) and ≤ 15° (control). Qualitative and quantitative assessments of MRI scans were performed. For quantitative assessment, receiver operating characteristic curves were used in order to determine the HVA cutoff with the highest accuracy.

**Results:**

A total of 66 MRI scans were included, 22 in the hallux valgus group and 44 in the control group. Wilcoxon signed-rank tests indicated a significant difference between the radiography and MRI measurements. Simple linear regression showed a nonlinear relationship between the measurements and values did not present a strong correlation. In comparison with the radiography measurements, MRI with an HVA cutoff of 16.4° exhibited the highest accuracy (86%). The accuracy of the subjective (qualitative) assessment was inferior to the objective assessment (measurement of the HVA).

**Conclusion:**

Hallux valgus can be diagnosed by measuring the HVA on MRI, satisfactory accuracy being achieved with an HVA cutoff of 16.4°.

## Abstract (pt)

**Objetivo:**

Avaliar a acurácia da ressonância magnética (RM) para o diagnóstico de hálux valgo usando radiografias com carga como padrão ouro.

**Materiais e Métodos:**

Análise retrospectiva de pacientes que realizaram RM do antepé e radiografias com carga, de janeiro a junho de 2015. O ângulo metatarsofalangiano (AMF) foi medido nas RMs e nas radiografias. O teste de Wilcoxon e regressão linear foram utilizados para comparar as mensurações. Pacientes foram divididos de acordo com os valores do AMF nas radiografias: > 15° (hálux valgo) e ≤ 15° (grupo controle). Avaliações qualitativa e quantitativa foram realizadas por RM. Para análise quantitativa, uma curva ROC foi utilizada para definir o ponto de corte com maior acurácia.

**Resultados:**

Foram incluídas 66 RMs, 22 no grupo com hálux valgo e 44 no grupo controle. O teste de Wilcoxon indicou diferença significativa entre os métodos. Avaliação de regressão demonstrou relação não linear entre as medidas e e os valores não apresentaram boa correlação. Considerando os grupos hálux valgo e controle, um valor de corte 16,4° na RM demonstrou maior acurácia (86%). A avaliação subjetiva foi inferior à avaliação objetiva.

**Conclusão:**

A medida do AMF na RM pode ser utilizada para diagnóstico de hálux valgo, com um valor de corte de 16,4°.

## INTRODUCTION

Hallux valgus is one of the most common afflictions of the foot, potentially causing pain and significant deformity^(1-3)^. The condition has a multifactorial, controversial etiology and is often associated with the use of inappropriate shoes, predisposing anatomy, and occupational risks, as well as genetic factors^(1-4)^. Hallux valgus occurs mainly among women and individuals over 60 years of age, with

Hallux valgus is defined as static subluxation of the first metatarsophalangeal joint, with lateral deviation of the toe and medial deviation of the first metatarsal bone. Several radiological parameters analyzed on radiography of the foot during weight bearing can be used to facilitate the diagnosis. Notable among such parameters is the hallux-metatarsophalangeal angle, or hallux valgus angle (HVA), which is defined as the angle formed by the intersection of the longitudinal axis of the first metatarsal bone diaphysis and the first proximal phalanx. Studies employing the gold standard (radiography of the foot during weight bearing) have established that HVA values ≤ 15° are normal^(5-9)^.

Because of its excellent contrast resolution, magnetic resonance imaging (MRI) is an excellent method for the assessment of conditions that affect the metatarsal region^(10)^. However, MRI scans are not acquired during weight bearing, and there are no objective MRI parameters for the diagnosis of hallux valgus. Considering the high prevalence of this condition and the occasional unavailability of radiographic studies during weight bearing, radiologists commonly extrapolate the criteria for radiography during weight bearing to MRI or even suggest the presence of hallux valgus on subjective grounds in MRI reports. Therefore, it is crucial to determine whether a criterion such as the HVA can be used in MRI.

In the present study, we assessed the accuracy of HVA measurement for the diagnosis of hallux valgus on MRI, using plain radiography under weight bearing as the gold standard. In addition, we assessed the accuracy of subjective analysis of MRI for the diagnosis of hallux valgus.

## MATERIALS AND METHODS

This was a retrospective analysis of MRI scans of the foot acquired at our hospital between January and June of 2015. We included only those scans that were accompanied by a plain radiograph of the foot under weight bearing that had been obtained within the preceding six months. Scans of patients subjected to any surgical procedure were excluded, as were those of patients presenting with fractures or other conditions affecting the hallux.

The study was approved by the local research ethics committee. Because the study had a retrospective design, the requirement for informed consent was waived.

### Plain radiography

Radiographs were obtained with a digital X-ray machine (Axiom Luminos; Siemens Medical Solutions, Erlangen, Germany), in compliance with the standards applied at our facility for radiography during weight bearing, which include frontal and lateral views, with the patient standing, and the following parameters^(7)^: 1.1 m focus-tofilm distance; 2.5 mAs; and 50 kV.

### Assessment of radiographs

Radiographs were assessed by two radiologists, one (designated the main examiner) with 5 years of experience in musculoskeletal radiology and one (designated the second examiner) with no such experience. The HVA was measured according to the guidelines formulated by the ad hoc committee of the American Orthopaedic Foot & Ankle Society on angular measurements^(7)^. The results reported by the main examiner were used for statistical analysis, and the results reported by the second examiner were used for assessment of interobserver agreement. We considered HVA values ≤ 15° to be normal. Measurements were recorded to one decimal place.

### MRI scans

The MRI examinations were performed in a variety of 1.5 T scanners-Aera (Siemens Medical Solutions); Espree (Siemens Medical Solutions); Avanto (Siemens Medical Solutions); and Optima 450W (GE Healthcare, Milwaukee, WI, USA)-and 3.0 T scanners-Achieva (Philips Medical Systems, Best, the Netherlands); Skyra (Siemens Medical Solutions); and HDX (GE Healthcare). All protocols were implemented as described in Table 1.

**Table 1:** Parameters used in the MRI sequences.

MRI parameter	MRI sequence				
	Sagittal fat-sat T2 (hallux)	Axial T1	Coronal fat-sat T2	Coronal T1	Axial fat-sat T2
FOV (mm)	120-140	130-140	120-140	120-140	130-140
TR (ms)	1820-2900	410-551	3800-4100	466-550	2200-2900
TE (ms)	2	9.4-11	37-42	10-9.9	39-42
Slice thickness (mm)	2.5-3.5	2.5-3.5	3.0-4.0	3.0-4.0	2.5-3.5
Spacing (cm)	0.3-0.4	0.3-0.4	0.3-0.4	0.3-0.4	0.3-0.4

### Quantitative assessment of MRI scans

The quantitative analysis of the MRI scans was performed by the same two radiologists who assessed the radiographs. The results reported by the main examiner were used for statistical analysis, and the results of the second examiner were used for assessment of interobserver agreement. The examiners were blinded to the plain radiography results at the time of assessment of MRI scans.

**Figure 1:**
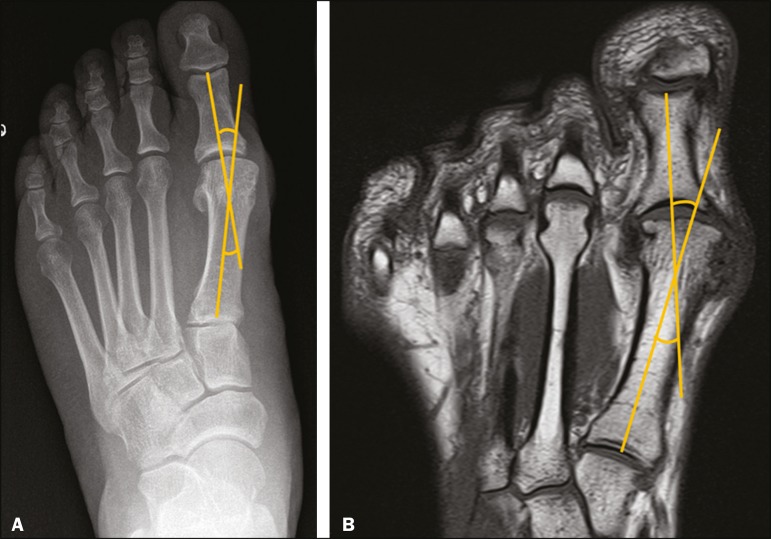
Technique for measurement of the HVA on plain radiography (**A**) and in a T1-weighted MRI sequence (**B**). The HVA was measured on MRI in a manner analogous to that recommended for plain radiography, based on two lines crossing the long axis of the diaphysis of the first metatarsal bone and the proximal phalanx of the hallux.

Angles were measured on T1-weighted sequences of the long axis of the foot, the measurements being analogous to the radiological measurements recommended in the guidelines formulated by the ad hoc committee of the American Orthopaedic Foot & Ankle Society on angular measurements^(7)^ , considering the angle formed by the largest longitudinal axis of the first metatarsal bone and the proximal phalanx of the hallux (Figure 1). Measurements were recorded to one decimal place.

### Qualitative assessment of MRI scans

A third examiner (with 17 years of experience in musculoskeletal radiology), who was blinded to the plain radiography results, assessed the MRI scans without using tools for the measurement of angles. This examiner classified the MRI scans of the foot dichotomously, as normal or as indicating hallux valgus.

### Statistical analysis

Measures of central tendency and dispersion were calculated for continuous variables, whereas absolute and relative frequencies were calculated for categorical variables. The assessment of diagnostic accuracy included analyses of sensitivity, specificity, positive predictive value, and negative predictive value, together with calculation of the corresponding 95% confidence intervals (95% CIs).

The Wilcoxon signed-rank test and simple linear regression were used in order to compare the measurement of the HVA on radiography during weight bearing and on MRI. To establish the optimal cutoff point for the HVA on MRI, the area under the receiver operating characteristic (ROC) curve was used. Interobserver agreement was assessed by calculating Cohen’s kappa. The diagnostic accuracy of the two methods tested (MRI assessed quantitatively and MRI assessed quantitatively) was determined by comparing their sensitivity and specificity with those of the gold standard (plain radiography during weight bearing).

## RESULTS

We evaluated 67 MRI scans of the foot accompanied by plain radiographs obtained within the preceding six months, corresponding to a total of 52 patients. One examination was excluded because the patient was diagnosed with gout with considerable hallux deformity. Therefore, 66 MRI scans from 51 patients were included in the analysis.

**Figure 2:**
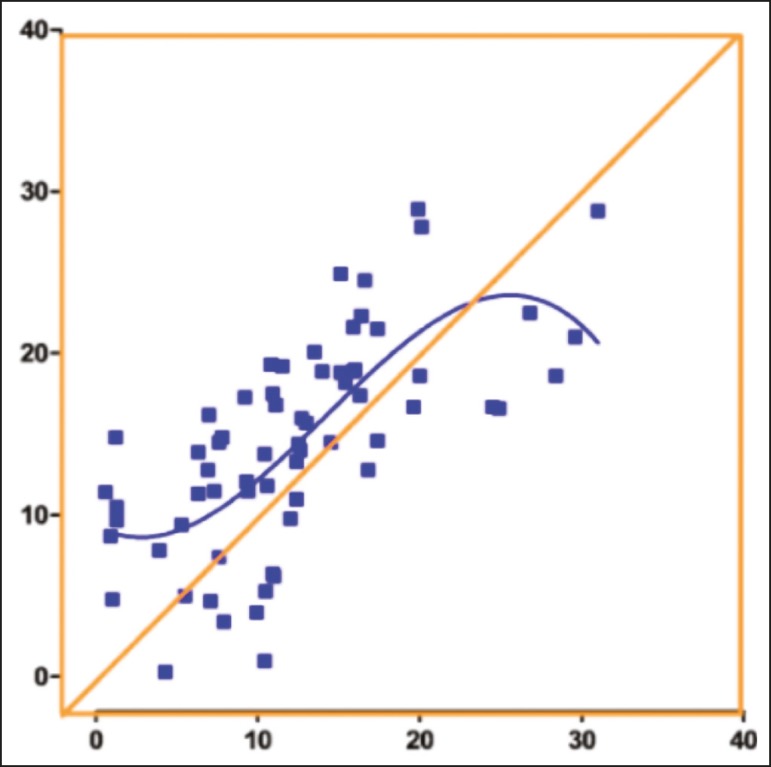
Simple linear regression comparing the HVAs measured on radiography and those measured on MRI. The scatterplot shows a nonlinear pattern.

The Wilcoxon signed-rank test (Table 2) indicated that the difference between the median HVA measured by MRI and that measured by radiography (14.55° and 11.30°, respectively) was significant (*p* = 0.004). A simple linear regression scatterplot shows that there was a nonlinear relationship between the two measurements (Figure 2). The two values did not present a strong correlation, even if we assume a linear pattern between the measurements (*r^2^* = 0.477). With a difference of 3.2 between the two medians, we were able to reject the null hypothesis that this response difference is zero with a probability (power) higher than 0.8. The type I error probability associated with that test is 0.05.

**Table 2:** MRI versus radiography (the gold standard) for the measurement of the HVA.

Method	HVA	
	Median (95% Cl)	P*
Radiography	11.3 (7.53-16.075)	0.004
MRI	14.55 (10.325-18.825)	

* Wilcoxon signed-rank test.

The cases were divided into two groups according to the HVA measurement on plain radiography during weight bearing. The group diagnosed with hallux valgus (HVA > 15°) comprised 22 examinations from 19 patients, with a mean age of 49.9 ± 14.5 years, of whom 13 (68%) were women. The group with a normal HVA (≤ 15°, control group) comprised 44 examinations from 35 patients, with a mean age of 43.5 ± 15.7 years, of whom 30 (66%) were women. Three patients who were tested bilaterally had one foot included in each group.

**Figure 3:**
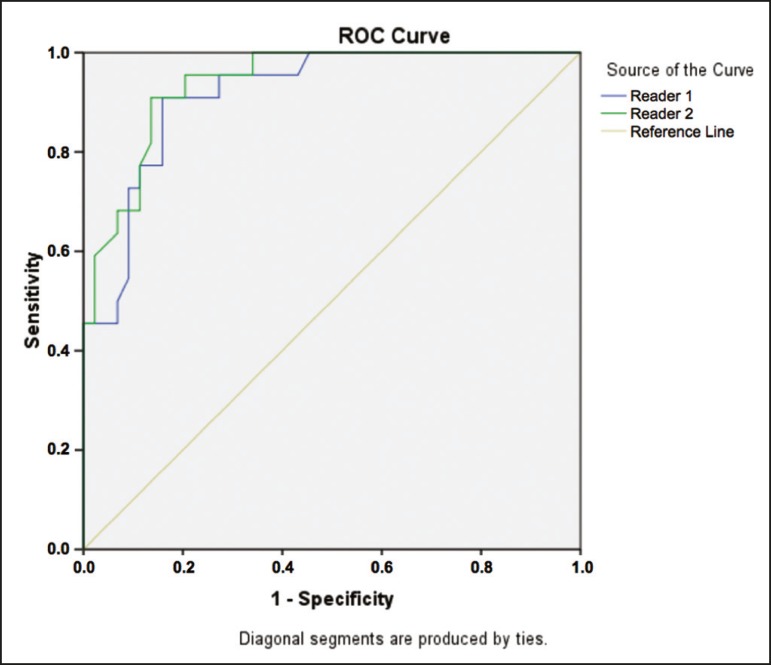
Plot showing the ROC curve for measurement of the HVA by two examiners (examiner 1: blue line; examiner 2: green line). An HVA of 16.4° was the cutoff point that exhibited the greatest accuracy (86.0%), with a sensitivity and specificity of 90.9% and 84.1%, respectively.

Regarding the HVA measurement, MRI exhibited satisfactory accuracy relative to the gold standard, with an area under the ROC curve > 0.9 (Figure 3) and a relevant asymptotic significance value (*p* < 0.0001), as shown in Table 3. An HVA cutoff point of 16.4° was established for the diagnosis of hallux valgus on MRI. An HVA > 16.4° was found to have an accuracy of 86%, a sensitivity of 90.9%, and a specificity of 84.1% (Table 4). If an HVA cutoff point of 15° were applied, as standardized for radiography during weight bearing, MRI would exhibit an accuracy of 81.8%, a sensitivity of 90.9%, and a specificity of 77.2%. The performance of the subjective (qualitative) assessment of the HVA was inferior to that of the objective (quantitative) assessment, with 86.4% sensitivity and 68.2% specificity for the diagnosis of hallux valgus compared with the gold standard (Table 5). The interobserver agreement for measurement of the HVA on MRI was excellent, with a kappa value of 0.91.

**Table 3:** Accuracy of MRI in comparison with that of radiography (the gold standard) for the measurement of the HVA.

Variable	AUC	SE	Asymptotic significance	Asymptotic 95% Cl
HVA on MRI (examiner 1)	0.92	0.03	< 0.0001	0.85-0.98
HVA on MRI (examiner 2)	0.94	0.03	< 0.0001	0.89-0.99

AUC, area under the ROC curve; SE, standard error.

**Table 4:** Comparison between MRI with an HVA cutoff of 16.4º and radiography (the gold standard) for the diagnosis of hallux valgus.

	Hallux valgus on radiography				
Hallux valgus on MRI	No (HVA ≤ 15º) n (%)	Yes (HVA > 15º) n (%)	P*	OR	95% Cl
No (HVA ≤ 16.4º)	37 (84.1%)	2 (9.1%)	<0.0001	52.9	10.1-278.8
Yes (HVA >16.4º)	7 (15.9%)	20 (90.9%)			

* Pearson’s chi-square; OR, odds ratio.

**Table 5:** Comparison between MRI assessed qualitatively and radiography (the gold standard) for the diagnosis of hallux valgus.

	Hallux valgus on radiography		
	No	Yes	
Hallux valgus on MRI	n (%)	n (%)	P*
No	30 (68.2%)	3 (13.6%)	< 0.0001
Yes	14 (31.8%)	19 (86.4%)	

* Pearson’s chi-square.

## DISCUSSION

The most relevant finding of the present study is that the diagnosis of hallux valgus is possible through measurement of the HVA on MRI, which exhibited satisfactory accuracy, sensitivity, and specificity values (86.0%, 90.9%, and 84.1%, respectively) when an HVA cutoff point of 16.4° was applied. Our study also assessed the ability to diagnose hallux valgus on MRI in a subjective manner, a situation similar to that found in clinical practice, in the absence of objective criteria. Even when performed by an experienced examiner, the subjective assessment was inferior to the objective assessment, exhibiting 86.4% sensitivity and 68.2% specificity.

Contrary to what we found in this study, two studies conducted by Heineman et al.^(11,12)^ demonstrated a good correlation between HVA measurements from MRI and those from radiography during weight bearing. Despite the differences, both studies established that the objective HVA measurement can be incorporated into clinical practice for the diagnosis of hallux valgus. Dessouky et al.^(13)^ also found a positive correlation between the two methods for the measurement of the HVA, although the strength of the correlation did not reach the level of statistical significance. Our data indicate that, in addition to exhibiting satisfactory accuracy, the objective HVA measurement was superior to the subjective assessment. In addition, the interobserver agreement between more and less experienced examiners for musculoskeletal tests was good, exhibiting satisfactory reproducibility.

Our data are relevant because of the widespread use of MRI to investigate pathologies of the foot, by orthopedic surgeons and, often, by nonspecialists. The results allow the radiologist to be active in reporting the hallux valgus findings and, often, to guide a physician unfamiliar with this diagnosis. In addition, MRI can access joint degeneration, hypertrophy (of bone and ligament), and injuries (of the plantar plate and sesamoid bone), which allow a global view of the disease and do not necessarily correlate with the imaging angle values^(14)^.

Our study has some limitations. First, because hallux valgus is a complex condition, the fact that we did not assess clinical data could be seen as a major limitation. In addition, although HVA measurement alone allows hallux valgus to be categorized as mild, moderate, or severe, we did not perform that stratification, because of the relatively small size of our sample. Furthermore, MRI and plain radiography were not performed on the same day. However, we believe that the impact of the time interval between the tests was minimized because we excluded patients who had fractures or had undergone surgery. Nevertheless, future studies could overcome these limitations by assessing the accuracy of MRI findings in association with the severity of hallux valgus, as established based on radiography, as well as by establishing correlations with clinical and surgical data.

## CONCLUSIONS

The present study showed that hallux valgus can be diagnosed objectively by MRI with an HVA cutoff point of 16.4°, and that objective measurement of the HVA exhibited satisfactory interobserver agreement and was found to be superior to subjective (qualitative) assessment.
